# Myasthenia Gravis: A Systematic Review

**DOI:** 10.7759/cureus.50017

**Published:** 2023-12-06

**Authors:** Aneesh K Mishra, Anuj Varma

**Affiliations:** 1 Medicine, Jawaharlal Nehru Medical College, Datta Meghe Institute of Higher Education and Research, Wardha, IND

**Keywords:** plasmapheresis, myasthenic crisis, thymectomy, neuromuscular junction, auto-antibody

## Abstract

Myasthenia gravis (MG), a rare disease, is the most common neuromuscular junction problem. It's the quintessential autoimmune disease with ocular, bulbar, respiratory, axial, and limb muscles exhibiting a typical fatigable weakening due to the development of antibodies against the acetylcholine receptor (AChR). Infections, stress, surgeries, thymus gland anomalies, and pharmaceutical side effects can also cause it. Ocular symptoms are initially experienced by most of the sufferers. The majority of the sufferers will go through at least one episode of symptom exacerbation during their illness. The immune system in MG interferes with nerve-muscle communication, causing muscles to become weak and tired quickly. The actual cause is not yet known, but a problem in the thymus gland may be the cause. In a person suffering from this disease, the size of the thymus becomes larger than normal, which is also called thymic hyperplasia. It is more common for women to have early-onset MG (EOMG) than for males to have late-onset MG (LOMG). Merely clinical evidence, encompassing the patients' medical history and physical indications of fluctuating muscle weakness in a specific region, is utilized to diagnose MG. Complementary diagnostic procedures and lab techniques aid in confirming the synaptic dysfunction and characterizing its kind and degree. Early diagnosis and the availability of effective treatments have reduced the burden of severe impairment and high mortality previously associated with MG. Current immunomodulation-based therapies come with side effects brought on by persistent immune suppression. Improved knowledge of this relatively uncommon but curable condition is required among primary carers. The objective of this review is to provide information about MG and to help people recognize its symptoms and start treatment without panic so that the progression of this disease can be stopped and complications can be avoided.

## Introduction and background

One of the most prevalent conditions that interfere with neuromuscular transmission is myasthenia gravis (MG). Its distinctive symptoms, which are mostly brought on by an immune response against the post-synaptic membrane of the neuromuscular junction (NMJ), include acute weakness and exhaustion, ocular muscles, bulbar functions, as well as limb and respiratory muscles [1]. Most people with MG initially experience ocular symptoms. At least one symptom aggravation will occur in most MG patients at some point during their disease [2]. In Asia, the prevalence of the disease is rising [3]. The disease is becoming more widely recognized in persons over 50. Autoimmune NMJ disorders can be diagnosed using various approaches, including imaging, pharmacological, electrophysiological, and serological antibody tests [4]. The clinical course of MG is significantly influenced by age and gender, so patient management of these variables requires special attention. The first year or two of the disease causes the greatest degree of weakness and high death rate; however, many patients recover after that [5]. Identification of the clinical features is essential for an early diagnosis. Delays of one to two years before diagnosis are not unusual due to the disease's low incidence in clinical practice and frequently undiagnosed symptoms [6]. Given its strong correlation with the severity of the disease, weariness is likely a contributing factor to the MG symptomatology [7]. MG comes in a variety of forms, some of which are listed below.

Based on the clinical presentation

Generalized MG

The hallmark of the uncommon autoimmune condition known as generalized MG is abnormal neural communication at the NMJ leading to weakness of the skeletal muscles. Muscle weakness affects not just the eyes but also other parts of the body [8].

Ocular MG

One subtype of MG called ocular MG affects only the muscles of the levator palpebrae superioris (LPS), orbicularis oculi, and extraocular areas. Ptosis can result in functional blindness and incarcerating diplopia in individuals with ocular MG. For the most part, medical care is needed [9].

Based on the genetic patterns

Congenital Vs Acquired MG

Mutations influencing pre or postsynaptic function at the neuromuscular synapse, leading to muscular weakness, are the cause of uncommon congenital myasthenic syndromes (CMS). A group of clinically diverse genetic disorders affecting the NMJ that start early are known as congenital myasthenic syndromes. Autoantibodies are responsible for the immune-mediated disease known as acquired MG. These antibodies are frequently directed against the acetylcholine receptors found at the NMJ [10].

Based on the antibody status

Seronegative Vs Seropositive MG

Individuals classified as seronegative do not produce antibodies against the acetylcholine receptor, and patients who do not produce both acetylcholine receptor and muscle-specific kinase antibodies are said to have double seronegative MG. The rare autoimmune condition known as seropositive MG impairs neuromuscular transmission. In this, antibodies are formed against acetylcholine receptors and muscle-specific kinase receptors [11].

Based on the age of onset

Juvenile Vs Adult MG 

When serum antibodies engage in interactions with nicotinic acetylcholine receptors located at the motor endplate muscle membrane, a chronic autoimmune disease known as juvenile MG develops, impairing neuromuscular transmission. Children aged below 18 years experience it. While myasthenia crisis deaths are uncommon, children are at a higher risk. A condition known as adult-onset MG occurs when it manifests after the age of 50. About 50% of individuals with adult-onset MG have antibodies to muscle titin; this is the main immunological difference between early-onset and late-onset MG [12].

Based on the placentally transferred antibodies

Transient Neonatal MG (TNMG)

A postsynaptic neuromuscular transmission defect known as TNMG affects 21% of infants born to mothers with active (and less often, remission) acquired MG. Neuromuscular transmission is hampered by maternal antibodies that are transferred transplacentally and target both receptors. Pathogenesis in infants without acetylcholine receptor antibodies is unknown [13].

## Review

Methodology

**Figure 1:**
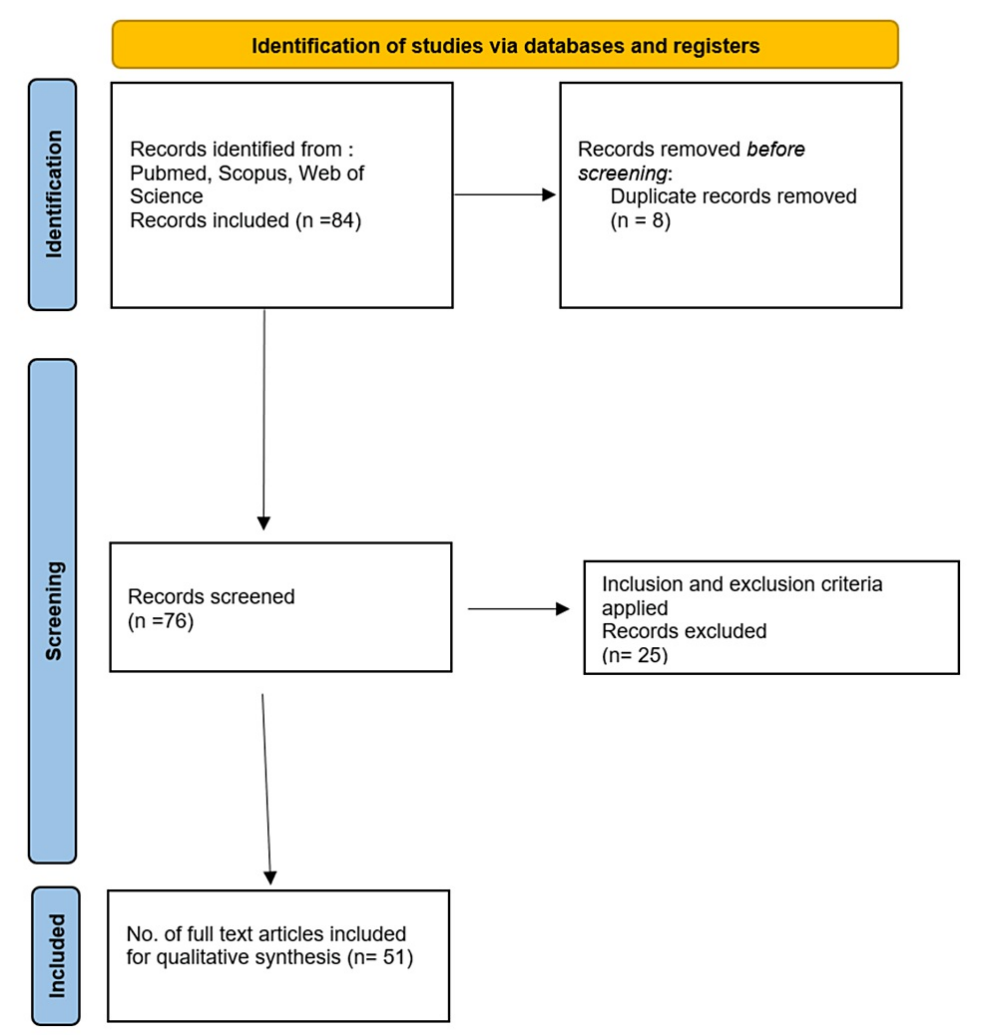
The process by which the study articles were selected.

A comprehensive search strategy was carefully implemented in this systematic review to find relevant articles from reputable databases including PubMed, Web of Science, and Scopus. A set of key terms and Medical Subject Headings (MeSH) terms, such as "Autoantibodies," and "Thymectomy," were used to cast a wide net. Only results pertaining to articles in English were used. In cases where multiple reports were released, the latest article from a related study was utilized. Research that involved animals, were published in languages other than English, or were irrelevant were excluded. After 84 articles were found in the initial search and were purged of duplicates, a two-stage screening procedure produced 76 articles that could be read in full. In the end, 51 studies were included in the review. The Preferred Reporting Items for Systematic Reviews and Meta-Analysis (PRISMA) flow diagram for searches is shown in Figure 1.

Pathogenesis of MG

Generation of Autoantibodies

Autoantibodies disrupt neuromuscular transmission by targeting the structural elements of the NMJ. The most often targeted proteins by pathogenic autoantibodies in MG are lipoprotein receptor-related protein-4 (LRP4), muscle-specific kinases, and nicotinic acetylcholine receptors. Immunogloulin-G1 and immunoglobulin-G3 antibodies against the acetylcholine receptor are the most common cause of these cases. Acetylcholine receptor turnover is accelerated and complement-mediated damage is inflicted, resulting in the postsynaptic membrane losing its acetylcholine receptors. Additionally, antibodies to muscle-specific kinase or similar proteins like agrin and LRP4 are detected. Muscle-specific kinase antibodies, primarily IgG4 in composition, interfere with the physiological role of muscle-specific kinase in maintaining synapses and adaptability, leading to disruption of the NMJ [14].

Role of Cytokines and T-cells

Pathogenic autoantibodies are produced and inflammation is induced at the NMJ by cytokines, T cells, B cells, and plasma cells that have been activated. Each of these processes is significant. There are several potential factors that could exacerbate the pathogenesis of MG. These include T helper type 17 (Th17) cells mediated chronic inflammation, follicular Th (Tfh) cells that stimulate B cells and plasma cells to produce autoantibodies and regulatory T (Treg) cell dysfunction that triggers the immune system. Higher proportions of Th1 and Th17 cells aggravate the pathophysiology of MG, despite the fact that higher proportions of Th2 and Treg cells slow down the disease progression [15].

Thymus Abnormalities

The pathophysiology of the disease undoubtedly involves the thymus. Production of autoantibodies seems to be concentrated in the thymus. For the most part, patients with anti-acetylcholine receptor antibodies have germinal centers, which are home to B cells that generate these antibodies. The MG thymus is considered an archetype for tertiary lymphoid neogenesis because of its modified form and function, which provides the perfect conditions for lymphocytes and antigen-presenting cells to interact and initiate an immune response. Roughly 75% of MG patients have impaired thymus function. Thymic tumors, or thymomas, affect 10% of individuals, but the thymus is "hyperplastic" in approximately 65% of cases and has active germinal centers [16].

Genetics

Around 35% of monozygotic MG twins are concordant, which suggests that environmental factors are primarily responsible for the disease's etiology. Nevertheless, MG is not subject to Mendelian inheritance. Family members of people with MG are approximately 1000 times more likely to have the illness than people in general [17]. Individuals suffering from MG have varied symptoms, and their level of muscle weakness fluctuates on a daily basis. Without treatment, symptoms typically worsen over time. Table 1 lists the MG characteristics [18].

**Table 1:** Clinical characteristics of myasthenia gravis (MG). Source: [18]

Ocular Manifestations	Bulbar Manifestations	Respiratory Manifestations
Ptosis, Diplopia, External ophthalmoplegia	Dysphagia, Dysarthria	Weak breathing, Respiratory failure
Orbicularis weakness, Incomitant strabismus	Holding up the head	Breathlessness
Blurred vision, Extraocular muscle palsies	Weakness, Fatigue	Muscle weakness

Specifications for diagnosing

Serological Tests

Acetylcholine receptor antibodies: These are the most prevalent and well-characterized antibodies in MG. By binding to and activating complement, which damages the postsynaptic membrane, by crosslinking with acetylcholine receptors, which speeds up their endocytosis and degradation, and by directly blocking the acetylcholine receptor binding sites, these antibodies disrupt transmission in the NMJ [19].

Muscle-specific kinase antibodies: It is rare for patients to have antibodies against the muscle acetylcholine receptor, instead most have antibodies against the muscle-specific kinase. Anti-muscle-specific kinase antibodies are absent in seropositive MG. These antibodies have been linked to more pronounced bulbar and neck weakness as well as increased respiratory distress in MG patients [20].

Pharmacological Tests

Edrophonium test (tensilon test): Acetylcholine levels in the NMJ are raised by the reversible acetylcholinesterase inhibitor edrophonium. It has a quick start and a brief duration of action. MG patients' skeletal and muscular strength temporarily improved due to the elevated acetylcholine levels in the NMJ. Edrophonium testing is rarely used but effective in cases of detectable ptosis. Testing with edrophonium was superior in patients with ocular MG [21].

Neostigmine test: It is a pharmacological test that shows MG patients have improved clinically. Intravenous neostigmine treatment is a fast, precise, and safe method for treating MG with ocular involvement in cases that are difficult to diagnose. To prevent false negative results, eyelid posture and strabismus are quantitatively assessed when administering the neostigmine test to MG patients with ocular involvement [22].

Ice pack test: It is an instantaneous and easy method to diagnose MG [23]. It is a relatively simple, affordable, and safe technique that the doctor can carry out right at the patient's bedside. The ice pack test also has no side effects and doesn't call for any pricey equipment or pharmaceuticals. This involves applying an ice pack to the patient's troublesome eye for a duration of three to five minutes. When the diplopia or ptosis improves, the response is favorable [24].

Electrodiagnosis

Repetitive nerve stimulation test (RNS): For diseases of the NMJ, including MG, the repeated nerve stimulation test is a helpful and often used neurophysiological test. Presynaptic nerve terminals that receive repeated electrical stimulation from a motor nerve gradually lose their capacity to release synaptic vesicles. As a result, the endplate potential of individuals with MG may drop below the cutoff point needed to produce action potentials in the muscles [25]. Since the findings of confirmation antibody testing frequently take a while to come back, rapid confirmation of the MG diagnosis can be facilitated by RNS in the inpatient setting [26].

Single-fiber electromyogram (SFEMG): The evaluation of individual muscle fiber action potentials (MFAPs) is possible through a highly selective technique known as single-fiber electromyography. SFEMG can detect paired jitter by stimulating motor nerve branches to particular end plates either voluntarily or through axonal stimulation [27]. The most exact clinical neurophysiological test is single-fiber electromyography [28].

Other Tests

Pulmonary function test (PFT): MG may affect the muscles of inspiration and expiration. Almost all MG patients have respiratory involvement, regardless of the clinical manifestations of the condition. An analysis of lung volume by Saraiva et al. revealed that all patients with generalized or ocular MG exhibited the myasthenic pattern [29].

Management

MG can not be cured; however, its symptoms can be managed and its immune system activity can be restrained. The immune system can be regulated using immunosuppressants and corticosteroids. These drugs aid in lowering the aberrant immunological response seen in MG. It is also possible to use cholinesterase inhibitors, such as pyridostigmine can also be used to enhance the transmission of signals between muscles and nerves.

Pyridostigmine Bromide

The majority of individuals with MG are advised to begin treatment with acetylcholinesterase inhibitors, such as pyridostigmine. Acetylcholinesterase inhibitors were made widely known as a therapy for MG by Dr. Mary Broadfoot Walker in 1934. Pyridostigmine helps to improve the transmission of nerve impulses at the NMJ by blocking the acetylcholinesterase enzyme from degrading acetylcholine [30].

Corticosteroids

One of the most popular immunosuppressive medications for immune-mediated illnesses like MG is corticosteroids. Reductions in inflammatory cytokine production and leukocyte-endothelial adherence have an extensive antagonistic impact on the immune response [31].

Azathioprine

The immunosuppressive drug that is most frequently used to cure MG is azathioprine [32]. Azathioprine inhibits DNA synthesis, so fewer new cells are produced. Azathioprine mainly inhibits the development of white blood cells. It lessens the generation of damaging autoantibodies by halting the growth of white blood cells, which are responsible for producing antibodies. Azathioprine is a viable substitute for corticosteroids in some myasthenic patients who require immunosuppression [33].

Mycophenolate Mofetil (MMF)

If conventional therapy regimens fail to treat MG, mycophenolate mofetil may be a helpful alternative. Its application is straightforward and typically well tolerated [34]. Adhesion molecule activity and glycosylation are inhibited by it, which reduces the recruitment of lymphocytes and monocytes to inflammatory areas [35].

Cyclosporine

Cyclosporin is an immunosuppressive medication authorized for MG patients with poor corticosteroid response following thymectomy. Cyclosporine prevents acetylcholine loss at the NMJ and nearly entirely inhibits antibody responses against acetylcholine receptors [36].

Cyclophosphamide

It is a DNA alkylating agent that significantly inhibits transcription and DNA replication activities and is efficient in refractory MG [37]. Using immunoablative dosages of cyclophosphamide for treatment causes a significant and long-lasting improvement in MG symptoms. Patients with unusually resistant diseases can benefit from high-dose cyclophosphamide [38].

Methotrexate (MTX)

Methotrexate, a potent immunosuppressive drug for autoimmune disorders, selectively inhibits dihydrofolate reductase and lymphocyte proliferation. MTX is taken into cells, where the glutamate fragment is added and retained as methotrexate polyglutamate (MTXPG) [39].

Intravenous (IV) therapies

Plasmapheresis

Patients with acutely deteriorating MG respond very well to plasmapheresis treatment [40]. Therapeutic plasma exchange, a form of extracorporeal blood purification, involves taking plasma out of the blood, and healthy donor plasma or albumin is used in its place. Immune complexes, pathogenic autoantibodies, toxins, and cryoglobulins are among the high molecular weight materials that are frequently extracted from plasma using this process. Its effectiveness in MG is attributed to eliminating autoimmune biologically active proteins, especially antibodies to the acetylcholine receptor, which dramatically benefits motor efficiency, endurance of muscles, and the NMJ's transmission [41].

Intravenous Immunoglobulin (IVIg)

Immunoglobulins gathered from thousands of donors make up this collection. IVIg is a substitute for many immunosuppressants [42]. A popular kind of treatment for several autoimmune neuromuscular illnesses is immunoglobulin therapy. It has been utilized for acute and long-term treatment of MG patients [43].

Monoclonal Antibody

Monoclonal antibody therapies for MG inhibit the complement's effector mechanism and neonatal crystallizable fragment receptor (FcRn) ability to lower antibody levels. Autoantibody synthesis has been decreased using antibodies against clusters of differentiate (CD20) and signaling pathways that promote lymphocyte activity [44].

Surgical management

Video-Assisted Thoracoscopic Surgery (VATS) Thymectomy

It is a form of surgery used to identify and address several disorders. Recently, it has developed into a very successful method for treating MG. The superior and radical VATS thymectomy minimizes access stress by removing all thymic tissue scattered in the cervical fat and anterior mediastinum. Compared to the open approach, there is also a lower risk of problems connected to the respiratory and cardiac systems [45].

Robot-Assisted Thoracoscopic Surgery (RATS) Thymectomy

Recent research has revealed that this procedure is safe and effective for treating tumors of the thymus and MG. Robotic surgery is becoming increasingly common, and its benefits include superior surgical vision and precise manipulation [46]. For anatomical reasons and in the case of MG patients who require extra care, a left-sided RATS thymectomy is preferable [47]. 

Complications of MG

Respiratory failure is a significant complication of MG. Myasthenia or cholinergic crises may have occurred due to treatment with a massive quantity of a cholinesterase inhibitor [48]. This term is used to describe the most severe type of MG. Bulbar symptoms and dysphagia are critical differential diagnoses for myasthenic crises. Myasthenic crisis is frequently triggered by lung infections, aspiration, sepsis, operative procedures, fast immune modulation tapering, start of corticosteroid treatment, and exposure to medications that can worsen myasthenic weakness [49]. Myasthenic crisis can be caused by weak respiratory muscles that lower tidal volumes, weak upper airway muscles that clog the airway and cause aspiration, or weak muscles in both the respiratory and the breathing systems [50]. The mainstays of the therapy include immunoglobulins, plasma exchange, and steroids. As a result of ever-increasing advances in neurocritical care, the mortality rate for MG has dropped to less than 5% [51]. Table 2 summarizes the various studies reviewed.

**Table 2:** Summary of the reviewed articles. CN-SFEMG: concentric-needle single-fiber electromyography

Serial no.	Authors name	Title of the article	Conclusions
1)	Herr et al. [3]	Population-based retrospective cohort study conducted in Taiwan on the rising incidence of generalized myasthenia gravis.	With rising prevalence rates and an increase in the involvement of older age groups, Taiwan's epidemiology of generalized myasthenia gravis is changing quickly, indicating a rising disease burden and rising healthcare expenses.
2)	Punga et al. [4]	The type, severity, and markers of neuromuscular junction diseases caused by the immune system.	Autoimmune neuromuscular junction disorders are rare. According to epidemiological research done over the past 5-10 years worldwide, there can be up to 29 cases of myasthenia gravis per million people each year when an antibody to the acetylcholine receptor is present. There are numerous ways to diagnose autoimmune neuromuscular junction disorders, including as imaging, pharmacological, electrophysiological, and serological antibody testing.
3)	Grob et al. [5]	Myasthenia gravis lasts a lifetime	Patient management of these variables demands special attention because age and gender played a significant role in the clinical course of myasthenia gravis. The first one to two years of the illness were the most severely affected, with a high death rate, following this time, many people began to recover.
4)	Ruitar et al. [7]	Fatigue in autoimmune myasthenia gravis: prevalence and related aspects	Given the robust correlation with the severity of the disease, weariness ought to be acknowledged as a component of myasthenia gravis symptomatology. However, this study shows that weariness is strongly correlated negatively with physically demanding activities in a sizable population of myasthenia gravis patients.
5)	Howard et al. [8]	Complement's role in myasthenia gravis at the neuromuscular junction.	Innate and antibody-mediated immunity both depend on complement, and complement activation and amplification create membrane attack complexes (MACs) are lipophilic proteins that induce membrane damage in cells. Both people and animal models with generalized myasthenia gravis have shown the involvement of complement.
6)	Cornblath et al. [9]	Handling myasthenia gravis in the ocular organs	There are numerous therapeutic options, such as thymectomy, intravenous immunoglobulin, pyridostigmine, immunosuppression, plasmapheresis lid crutches, and surgery on the muscles surrounding the eyes for ptosis.
7)	Gilhus et al. [10]	Myasthenia gravis and congenital myasthenic syndromes are two conditions	Muscle weakness is the hallmark of myasthenia gravis, an autoimmune disease caused by antibodies that target components of the postsynaptic membrane. Muscle deficit is the result of uncommon mutations altering pre- or postsynaptic function at the neuromuscular synapse, which causes congenital myasthenic disorders.
8)	Vincent et al. [11]	Myasthenia gravis that is seronegative	Certain individuals with myasthenia gravis are referred to as "seronegative" (SNMG) if they do not exhibit any detectable acetylcholine receptor (AChR) antibodies.
9)	Li et al. [19]	Clinical importance of a serological diagnosis of myasthenia gravis	Antibody tests are now even more important than before in the diagnosis of myasthenia gravis because of the identification of new antigens in the disease and the creation of assays with higher sensitivity.
10)	Sciacca et al. [22]	Assessment of neostigmine test in myasthenia gravis by clinical and CN-SFEMG methods	A trustworthy method for assessing MG patients' reaction to neostigmine acute dosing is concentrated needle single fiber electromyography, or CN-SFEMG. Furthermore, subclinical improvement in ocular MG may be demonstrated by neurophysiological NT alterations, surpassing the clinical scale.
11)	Yamamoto et al. [23]	Effect of local chilling on myasthenic muscle's excitation-contraction coupling: An additional way to understand the myasthenia gravis ice-pack test	When myasthenic muscle is cooled, two things can happen. Electrical synaptic transmission at the endplate is primarily impacted by two factors: a transient effect and a persistent effect on E-C coupling in the muscle. Myasthenia gravis's impaired E-C coupling gradually improves with the ice-pack test.
12)	Kim et al. [25]	Based on the outcomes of a first repeated nerve stimulation test, generalized myasthenia gravis is expected to develop from ocular myasthenia gravis.	When used on limb muscles, the RNS test can help predict the conversion to generalized MG in patients with ocular start.
13)	Saraiva et al. [29]	An evaluation of the myasthenia gravis patients' respiratory systems. A crucial instrument for identifying the disease's clinical signs and diagnosing it	Myasthenic gravis might impair the muscles of the expiratory and inspiratory phases. Patients in their male ocular form acted in a more benign manner in terms of lung function, only displaying the myasthenic pattern in the presence of lung impairment.
14)	Remijn-Nelissen et al. [30]	In a cross-sectional study, the efficacy and adverse effects of pyridostigmine in the treatment of myasthenia gravis were examined	The majority of international guidelines suggest starting the therapy of myasthenia gravis with pyridostigmine. Pyridostigmine is still a medication with a very good long-term safety record and is easily accessible at a reasonable price.
15)	Lotan et al. [31]	What proof is there that corticosteroid medication causes myasthenia gravis to worsen? An organised analysis	One of the most popular immunosuppressive drugs for immune-mediated diseases, such as myasthenia gravis is corticosteroids (CS).
16)	Witte et al. [33]	Using azathioprine to treat myasthenia gravis	When immunosuppressive myasthenic patients are needed, azathioprine is an acceptable substitute for corticosteroids.
17)	Schneider et al. [34]	Treating severe myasthenia gravis with mycophenolate mofetil	When conventional therapy regimens fail to alleviate severe myasthenia gravis, mycophenolate mofetil may be a valuable substitute. Its application is straightforward and it is generally well-tolerated.
18)	Pasnoor et al. [39]	Phase II trial of methotrexate in individuals with myasthenia gravis	Methotrexate is a useful immunosuppressive drug for autoimmune disorders since it selectively inhibits the activity of dihydrofolate reductase and lymphocyte proliferation.
19)	Kuks et al. [40]	using plasmapheresis to treat myasthenia gravis. an examination	As a means of controlling symptoms until more effective types of treatment are developed, plasmapheresis is an important part of the management of extremely unwell or disabled myasthenia gravis patients.
20)	Park et al. [46]	Thoracic surgery with robot assistance: thymectomy	As a secure and efficient treatment for thymic tumors and myasthenia gravis, robotic thymectomy has grown in favor recently.
21)	Juel et al. [50]	Handling myasthenic crises and providing preoperative care in myasthenia gravis	As long as patients receive proper treatment and prompt respiratory care to lessen upper airway and respiratory muscle myasthenic weakness, myasthenic crises shouldn't be fatal.

## Conclusions

MG is a relatively uncommon illness that destroys the communication between the nervous system and the muscles. A lack of specific essential molecules for the body brings on this illness. When someone is affected by it, they become exhausted. This disease can strike at any age, even in childhood, but older males over 60 and young adult women under 40 are more at risk. An individual may need to undergo specific physical examinations and several other tests to confirm the precise origin of the disease, depending on the symptoms, infectious diseases, and different past medical histories. MG is a difficult ailment that can range in severity and have an impact on all facets of life. MG has no treatment options and it is managed with the help of medicine, plasmapheresis, thymus gland removal, IVIG, and rest to reduce muscle weakness. Treatment aims to manage symptoms and modulate immune system activity. Individuals can manage symptoms and have fulfilling lives with the help of appropriate medical care, emotional support, and lifestyle changes. Early diagnosis is crucial to the management of this illness. While avoiding eating and breathing issues, the treatment aims to improve general muscle function. In most cases, people with MG can regain muscle strength and live ordinary or near-ordinary everyday lives. There are emerging new therapies that target particular immune pathways. Future treatments may benefit from research into gene-based medicine.

## References

[1] Martínez Torre S, Gómez Molinero I, Martínez Girón R (2018). An update on myasthenia gravis [Article in Spanish]. Semergen.

[2] Hehir MK, Silvestri NJ (2018). Generalized myasthenia gravis: classification, clinical Presentation, natural History, and epidemiology. Neurol Clin.

[3] Herr KJ, Shen SP, Liu Y, Yang CC, Tang CH (2023). The growing burden of generalized myasthenia gravis: a population-based retrospective cohort study in Taiwan. Front Neurol.

[4] Punga AR, Maddison P, Heckmann JM, Guptill JT, Evoli A (2022). Epidemiology, diagnostics, and biomarkers of autoimmune neuromuscular junction disorders. Lancet Neurol.

[5] Grob D, Brunner N, Namba T, Pagala M (2008). Lifetime course of myasthenia gravis. Muscle Nerve.

[6] Koch JA, Steele MR, Koch LM (2013). Myasthenia gravis. J Gerontol Nurs.

[7] Ruiter AM, Verschuuren JJ, Tannemaat MR (2021). Prevalence and associated factors of fatigue in autoimmune myasthenia gravis. Neuromuscul Disord.

[8] Howard JF Jr (2018). Myasthenia gravis: the role of complement at the neuromuscular junction. Ann N Y Acad Sci.

[9] Cornblath WT (2018). Treatment of ocular myasthenia gravis. Asia Pac J Ophthalmol (Phila).

[10] Gilhus NE (2023). Myasthenia gravis and congenital myasthenic syndromes. Handb Clin Neurol.

[11] Vincent A, McConville J, Farrugia ME, Newsom-Davis J (2004). Seronegative myasthenia gravis. Semin Neurol.

[12] Papazian O, Alfonso I (2009). Juvenile myasthenia gravis [Article in Spanish]. Medicina (Buenos Aires).

[13] Papazian O (1992). Transient neonatal myasthenia gravis. J Child Neurol.

[14] Fichtner ML, Jiang R, Bourke A, Nowak RJ, O'Connor KC (2020). Autoimmune pathology in myasthenia gravis disease subtypes is governed by divergent mechanisms of immunopathology. Front Immunol.

[15] Uzawa A, Kuwabara S, Suzuki S (2021). Roles of cytokines and T cells in the pathogenesis of myasthenia gravis. Clin Exp Immunol.

[16] Weiss JM, Cufi P, Le Panse R, Berrih-Aknin S (2013). The thymus in autoimmune myasthenia gravis: paradigm for a tertiary lymphoid organ. Rev Neurol (Paris).

[17] Avidan N, Le Panse R, Berrih-Aknin S, Miller A (2014). Genetic basis of myasthenia gravis - a comprehensive review. J Autoimmun.

[18] Gwathmey KG, Burns TM (2015). Myasthenia gravis. Semin Neurol.

[19] Li Y, Peng Y, Yang H (2023). Serological diagnosis of myasthenia gravis and its clinical significance. Ann Transl Med.

[20] Lazaridis K, Tzartos SJ (2020). Autoantibody specificities in myasthenia gravis; implications for improved diagnostics and therapeutics. Front Immunol.

[21] Pasnoor M, Dimachkie MM, Farmakidis C, Barohn RJ (2018). Diagnosis of myasthenia gravis. Neurol Clin.

[22] Sciacca G, Reggio E, Mostile G, Nicoletti A, Drago F, Salomone S, Zappia M (2018). Clinical and CN-SFEMG evaluation of neostigmine test in myasthenia gravis. Neurol Sci.

[23] Yamamoto D, Imai T, Tsuda E (2017). Effect of local cooling on excitation-contraction coupling in myasthenic muscle: another mechanism of ice-pack test in myasthenia gravis. Clin Neurophysiol.

[24] Almeida DF, Radaeli Rde F, Melo AC Jr (2008). Ice pack test in the diagnosis of myasthenia gravis. Arq Neuropsiquiatr.

[25] Kim KH, Kim SW, Shin HY (2021). Initial repetitive nerve stimulation test predicts conversion of ocular myasthenia gravis to generalized myasthenia gravis. J Clin Neurol.

[26] Clifford KM, Wu CK, Post D, Shaik R, Muppidi S (2023). Utility of repetitive nerve stimulation in the diagnosis of myasthenia gravis in the inpatient setting. Neurohospitalist.

[27] Juel VC (2019). Single fiber electromyography. Handb Clin Neurol.

[28] Kouyoumdjian JA, Stålberg EV (2007). Concentric needle single fiber electromyography: normative jitter values on voluntary activated extensor digitorum communis. Arq Neuropsiquiatr.

[29] Saraiva PA, de Assis JL, Marchiori PE (1996). Evaluation of the respiratory function in myasthenia gravis. An important tool for clinical feature and diagnosis of the disease. Arq Neuropsiquiatr.

[30] Remijn-Nelissen L, Verschuuren JJ, Tannemaat MR (2022). The effectiveness and side effects of pyridostigmine in the treatment of myasthenia gravis: a cross-sectional study. Neuromuscul Disord.

[31] Lotan I, Hellmann MA, Wilf-Yarkoni A, Steiner I (2021). Exacerbation of myasthenia gravis following corticosteroid treatment: what is the evidence? A systematic review. J Neurol.

[32] Lorenzoni PJ, Kay CS, Zanlorenzi MF, Ducci RD, Werneck LC, Scola RH (2020). Myasthenia gravis and azathioprine treatment: adverse events related to thiopurine S-methyl-transferase (TPMT) polymorphisms. J Neurol Sci.

[33] Witte AS, Cornblath DR, Parry GJ, Lisak RP, Schatz NJ (1984). Azathioprine in the treatment of myasthenia gravis. Ann Neurol.

[34] Schneider C, Gold R, Reiners K, Toyka KV (2001). Mycophenolate mofetil in the therapy of severe myasthenia gravis. Eur Neurol.

[35] Allison AC, Eugui EM (2000). Mycophenolate mofetil and its mechanisms of action. Immunopharmacology.

[36] Drachman DB, Adams RN, McIntosh K, Pestronk A (1985). Treatment of experimental myasthenia gravis with cyclosporin A. Clin Immunol Immunopathol.

[37] Gomez-Figueroa E, Garcia-Trejo S, Bazan-Rodriguez L, Cervantes-Uribe R, Chac-Lezama G, López-Hernández JC, Vargas-Cañas S (2020). Intravenous cyclophosphamide monthly pulses in refractory myasthenia gravis. J Neurol.

[38] Lin PT, Martin BA, Weinacker AB, So YT (2006). High-dose cyclophosphamide in refractory myasthenia gravis with MuSK antibodies. Muscle Nerve.

[39] Pasnoor M, He J, Herbelin L, Dimachkie M, Barohn RJ (2012). Phase II trial of methotrexate in myasthenia gravis. Ann N Y Acad Sci.

[40] Kuks JB, Skallebaek D (1998). Plasmapheresis in myasthenia gravis. A survey. Transfus Sci.

[41] Kumar R, Birinder SP, Gupta S, Singh G, Kaur A (2015). Therapeutic plasma exchange in the treatment of myasthenia gravis. Indian J Crit Care Med.

[42] Peng X, Xie XB, Tan H (2022). Effects of plasma exchange combined with immunoglobulin therapy on consciousness, immune function, and prognosis in patients with myasthenia gravis crisis: a prospective randomized test. Comput Math Methods Med.

[43] Bourque PR, Pringle CE, Cameron W, Cowan J, Chardon JW (2016). Subcutaneous immunoglobulin therapy in the chronic management of myasthenia gravis: a retrospective cohort study. PLoS One.

[44] Alabbad S, AlGaeed M, Sikorski P, Kaminski HJ (2020). Monoclonal antibody-based therapies for myasthenia gravis. BioDrugs.

[45] Qi K, Wang B, Wang B, Zhang LB, Chu XY (2016). Video-assisted thoracoscopic surgery thymectomy versus open thymectomy in patients with myasthenia gravis: a meta-analysis. Acta Chir Belg.

[46] Park S (2021). Robot-assisted thoracic surgery thymectomy. J Chest Surg.

[47] Li F, Ismail M, Elsner A, Uluk D, Bauer G, Meisel A, Rueckert JC (2019). Surgical techniques for myasthenia gravis: robotic-assisted thoracoscopic surgery. Thorac Surg Clin.

[48] Hetherington KA, Losek JD (2005). Myasthenia gravis: myasthenia vs. cholinergic crisis. Pediatr Emerg Care.

[49] Stetefeld HR, Schroeter M (2018). Myasthenic crisis [Article in German]. Fortschr Neurol Psychiatr.

[50] Juel VC (2004). Myasthenia gravis: management of myasthenic crisis and perioperative care. Semin Neurol.

[51] Godoy DA, Mello LJ, Masotti L, Di Napoli M (2013). The myasthenic patient in crisis: an update of the management in neurointensive care unit. Arq Neuropsiquiatr.

